# Supplementary material for: Poly(l-Ornithine)-Based Polymeric Micelles as pH-Responsive Macromolecular Anticancer Agents
Source: Pharmaceutics. 2023 Apr 21;15(4):1307. doi: 10.3390/pharmaceutics15041307 (PMC10143059; doi:10.3390/pharmaceutics15041307)
Supplement: Supplementary file 1 [file pharmaceutics-15-01307-s001.zip › pharmaceutics-2344798-Supplementary.pdf]

pharmaceutics

Supplementary Materials: Poly(L-ornithine)-based Polymeric Micelles as pH-responsive Macromolecular Anticancer Agents

Miao Pan, Chao Lu, Wancong Zhang, Huan Huang, Xingyu Shi, Shijie Tang and Daojun Liu

Supplementary Materials

Materials

δ-Benzyloxycarbonyl-L-ornithine and L-phenylalanine were supplied by GL Biochem (Shanghai, China). Hexylamine (99%) and 1,2-dicarboxylic-cyclohexene anhydride (DCA) were obtained from Aladdin Biochemical Technology Co. (Shanghai, China). Triphosgene and hydrogen bromide in acetic acid (33% *w*/*w*) were purchased from TCI Development Co. (Shanghai, China). Dichloromethane (DCM), ethyl acetate, petroleum ether, tetrahydrofuran (THF), and dimethyl sulfoxide (DMSO) were purchased from Sigma-Aldrich (Singapore) and were dried prior to use. Dulbecco's modified Eagle's medium (DMEM), alamarBlue and lactate dehydrogenase (LDH) assay kits were bought from Invitrogen (Singapore). 3,3′-Dioctadecyloxacarbocyanine perchlorate (DiO), propidium iodide (PI), calcein acetoxymethyl ester (calcein-AM), bisbenzimide (Hoechst) and an Annexin V-FITC Apoptosis Detection kit were purchased from Meilun Biotechnology Co. (Dalian, China). Dialysis tubes with molecular weight cut-offs of 1.0, 2.0, and 3.5 kDa were supplied by Viskase (Darien, IL).

Human luminal breast cancer cells (MCF-7), human breast ductal carcinoma cells (BT474), human non-small cell lung cancer cells (A549), human cervical cancer cells (HeLa), human normal liver cells (LO2), and human renal tubular epithelial cells (HK-2) were obtained from ATCC. Human hepatocellular carcinoma cells (HepG2) were provided by Professor Wenxiu Ni from the Department of Pharmacy, Shantou University Medical College, China. DOX-resistant MCF-7/ADR cells were kindly provided by Dr. Yiyan Yang from The Nanos, Institute of Bioengineering and Nanotechnology, Singapore.

Synthesis and characterization of polypeptide

δ-Benzyloxycarbonyl-L-ornithine *N*-carboxyanhydride (ZLO-NCA) and L-phenylalanine *N*-carboxyanhydride (LF-NCA) were prepared upon phosgenation of ZLO and ZLF in anhydrous ethyl acetate and anhydrous THF, respectively, following our previously reported method.[36] Hexylamine was employed to initiate the sequential ring-opening polymerization of ZLO-NCA and LF-NCA to produce PZLO-*b*-PLF, as illustrated in Scheme 1. The produced $PZLO_{30}$ (**PZ1**), $PZLO_{30}$-*b*-PLF_{4} (**PZ2**), $PZLO_{30}$-*b*-PLF_{8} (**PZ3**), and $PZLO_{30}$-*b*-PLF_{12} (**PZ4**) were characterized by ^1H NMR in DMSO-d_6 and their molecular weights were measured by GPC using Shodex KD802.5 and KD804 columns and a 2414 RI detector. DMF was employed as the eluent at a flow rate of 1 mL/min and the molecular weights were calibrated using poly(methyl methacrylate) (PMMA) standards.

In the second step, the benzyloxycarbonyl groups in the side chains of PZLO were deprotected in HBr/CH_3COOH to prepare the final products PLO_{30} (**P1**), PLO_{30}-*b*-PLF_{4} (**P2**), PLO_{30}-*b*-PLF_{8} (**P3**), and PLO_{30}-*b*-PLF_{12} (**P4**) following our previously reported method.[43] The final products were obtained by dialyzing thoroughly against deionized water followed by lyophilization. The final products were characterized by ^1H NMR in D_2O and their molecular weights were measured by GPC using an Ultrahydrogel linear column and a 2414 RI detector. HAc–NaAc buffer (0.5 M, pH 4.5) was used as the eluent at a flow rate of 1 mL min^{-1}, and the molecular weights were calibrated using poly(ethylene glycol) (PEG) standards.

pH-sensitive copolypeptides PLO(DCA)-*b*-PLF were obtained by modification of the side chains of PLO in PLO-*b*-PLF with DCA.[46] Briefly, PLO-*b*-PLF was dissolved in 0.2 M bicarbonate buffer at pH 9.2. DCA powder (1.5 eq to primary amino groups of PLO-*b*-PLF) was then added slowly to the polymer solution where the pH of the solution was kept at approximately 9.0 by the addition of 1 M NaOH. The solution was stirred at 4°C for 1.5 h, followed by the sequential 2-h dialyses against 0.1 M, 0.05 M, 0.01 M, 0.005 M bicarbonate buffer and deionized water. The final products were obtained following lyophilization and characterized by ^{1}H NMR in D_2O.

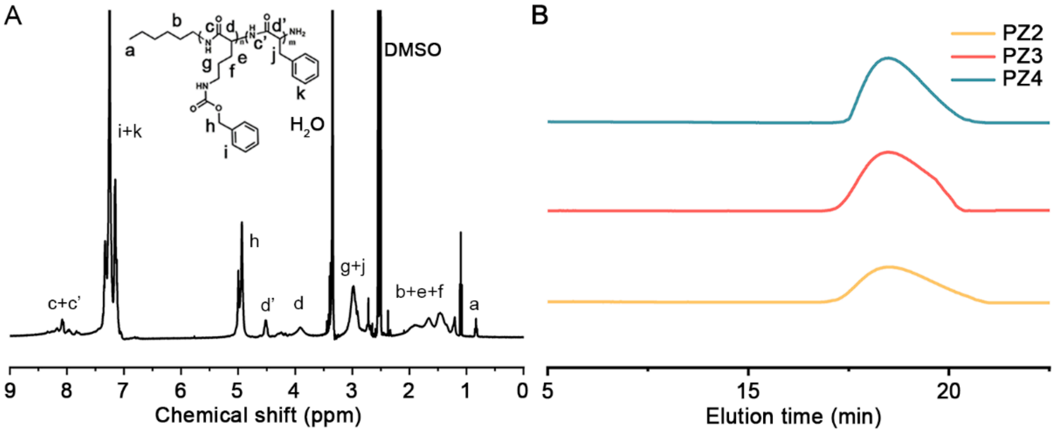

Figure S1. Representative ^{1}H NMR spectrum (**A**) and GPC chromatograms (**B**) of PZLO-*b*-PLF.

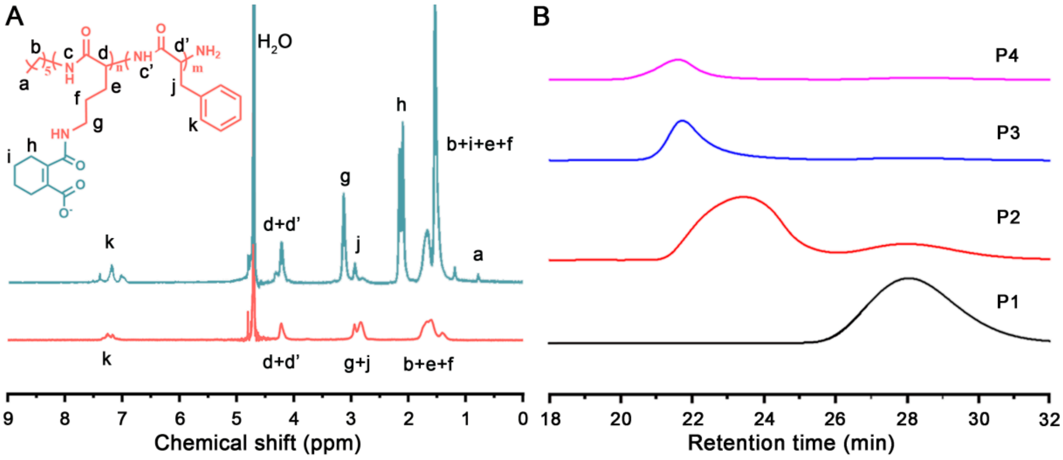

Figure S2. Representative ^{1}H NMR spectra (**A**) of PLO-*b*-PLF (red) and PLO(DCA)-*b*-PLF (blue) in D_2O, and GPC chromatograms (**B**) of PLO-*b*-PLF.

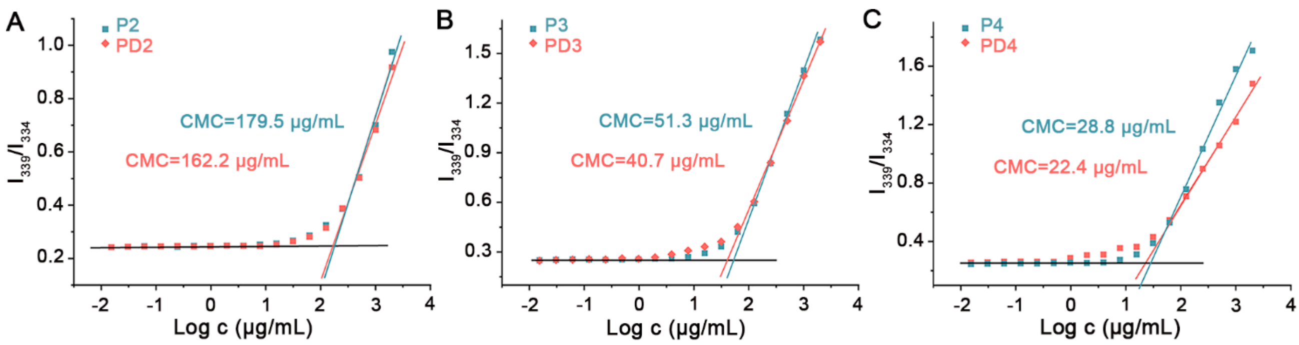

Figure S3. Plot of the I_{339}/I_{334} of pyrene vs. log(*c*) of (**A**) **P2** and **PD2**, (**B**) **P3** and **PD3**, and (**C**) **P4** and **PD4**. The CMCs of **P2**, **P3**, and **P4** were determined in PBS at pH 7.4, whereas the CMCs of **PD2**, **PD3**, and **PD4** were determined in bicarbonate buffer at pH 9.2.

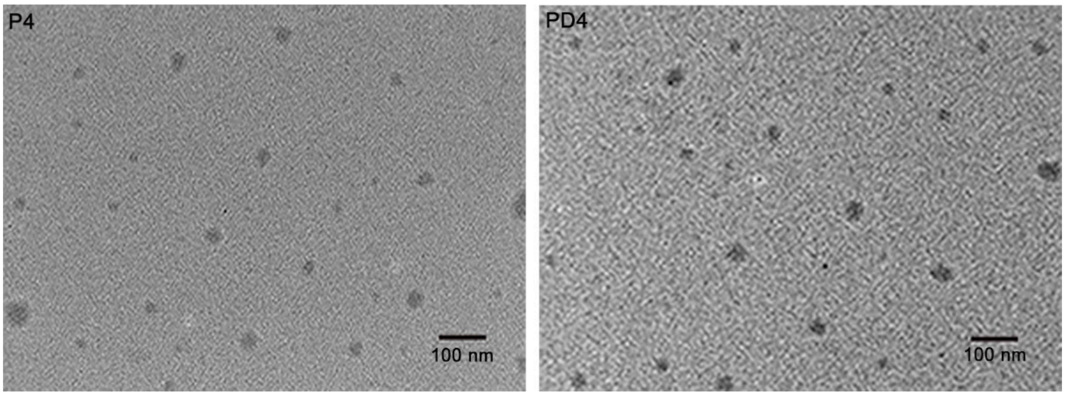

Figure S4. TEM images of polymeric micelles formed from **P4** and **PD4**.

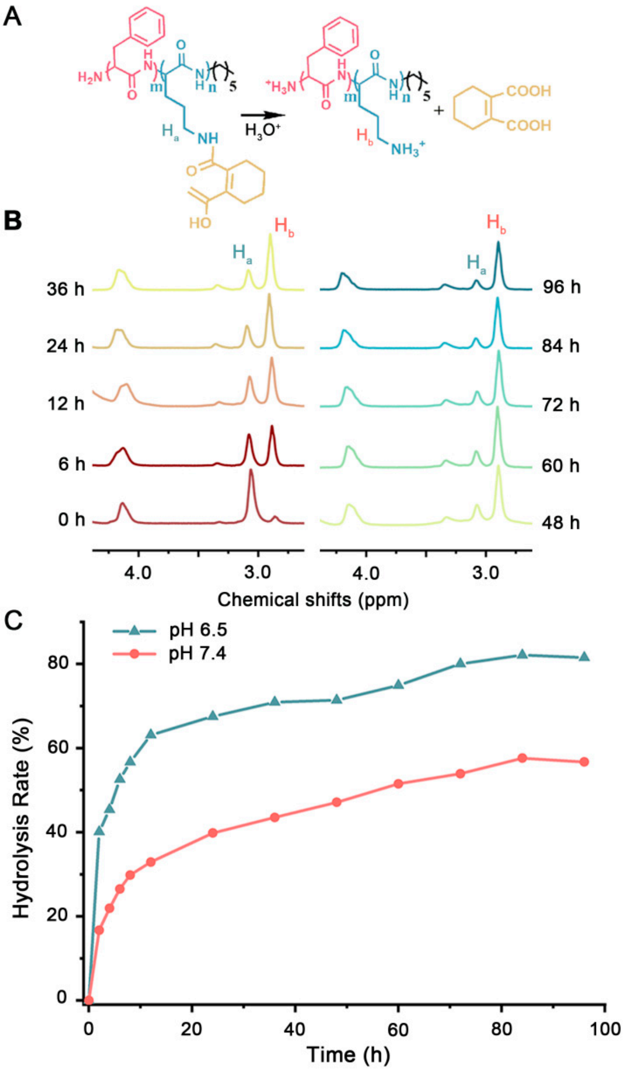

Figure S5. (**A**) Hydrolysis scheme of **PD4**; (**B**) ^{1}H NMR spectra of PLO(DCA) as a function of incubation time at pH 6.5; (**C**) hydrolysis kinetics of PLO(DCA) at pH 6.5 and pH 7.4.

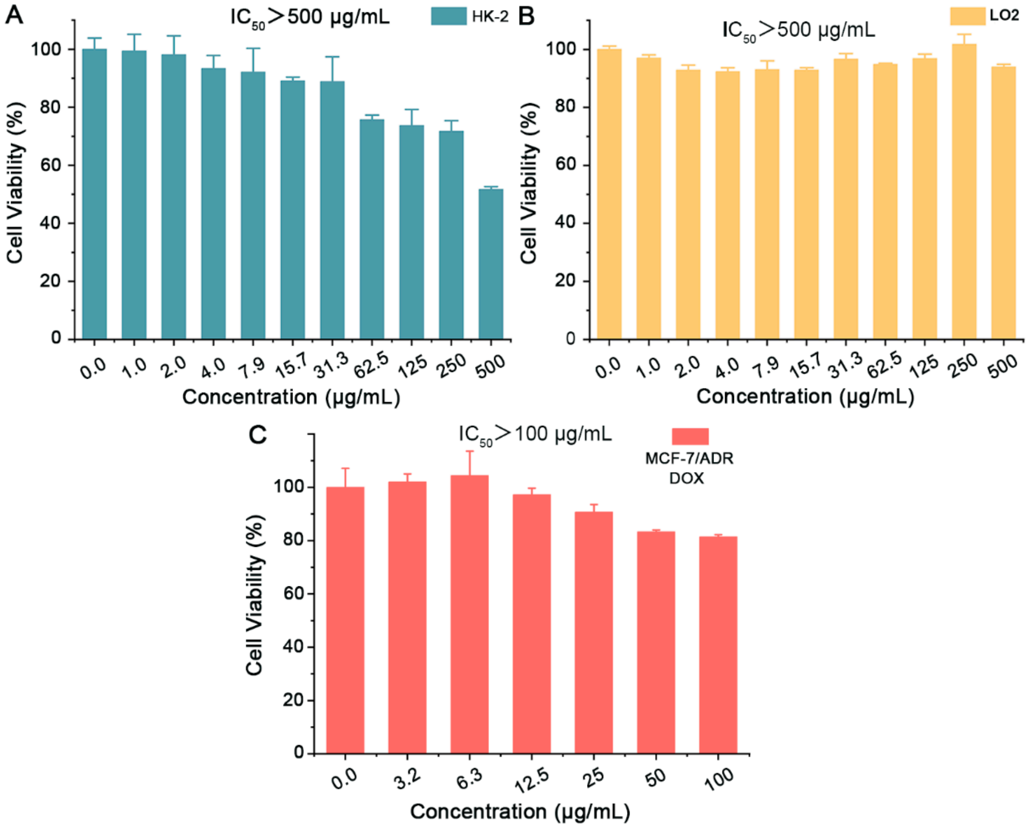

Figure S6. Viability of (**A**) HK-2 cells, (**B**) LO2 cells after incubation with different concentrations of **PD4** for 24 h at pH 7.4. as well as (**C**) MCF-7/ADR cells after incubation with different concentrations of DOX for 24 h.

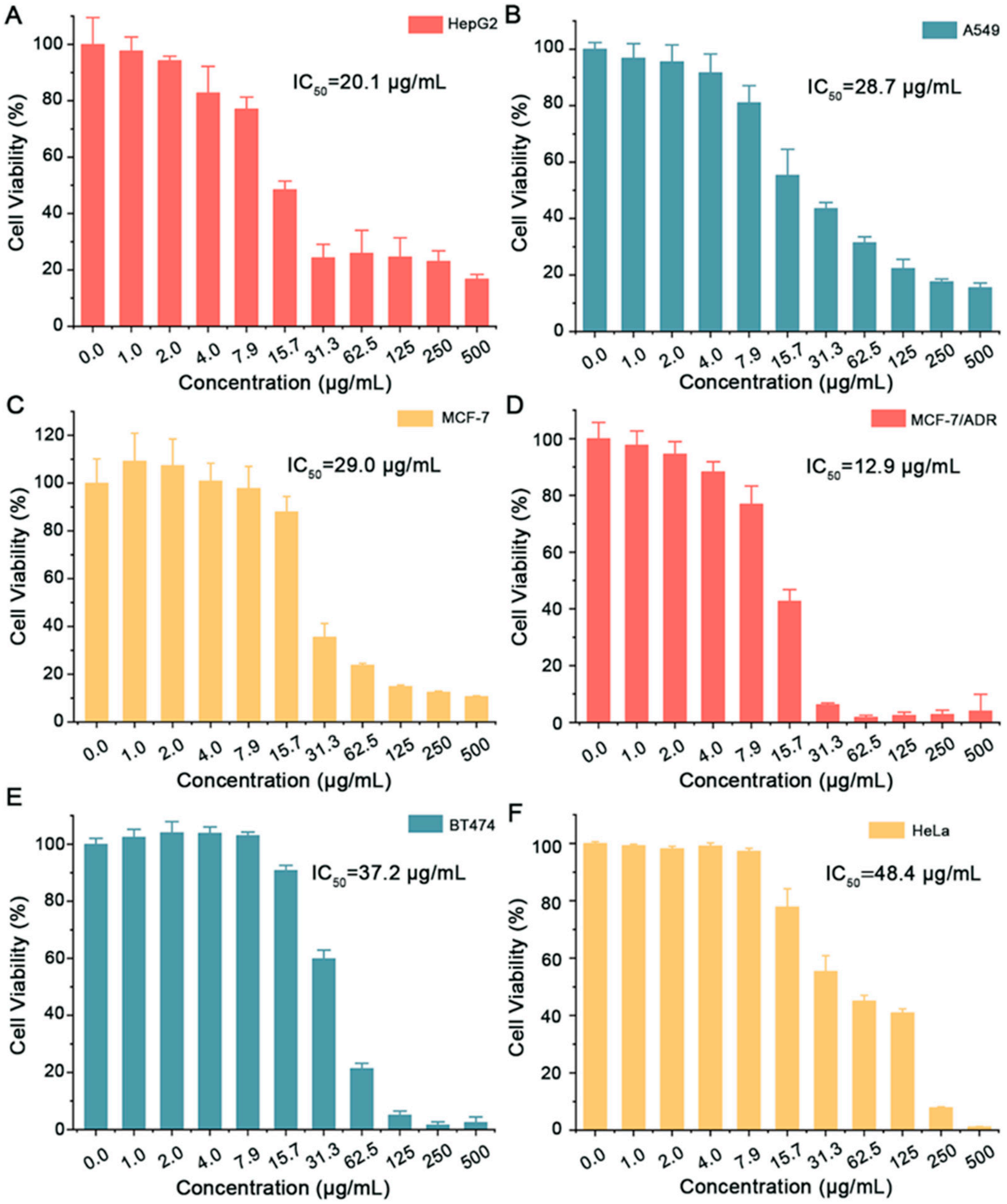

Figure S7. (**A**–**F**) Viability of different cancer cells after incubation with different concentrations of **PD4** for 24 h at pH 6.5.

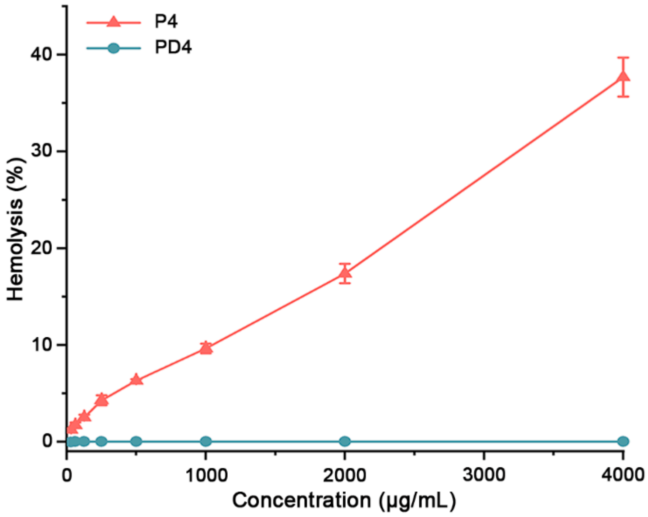

Figure S8. Hemolysis of **P4** and **PD4** as a function of their concentrations at pH 7.4.

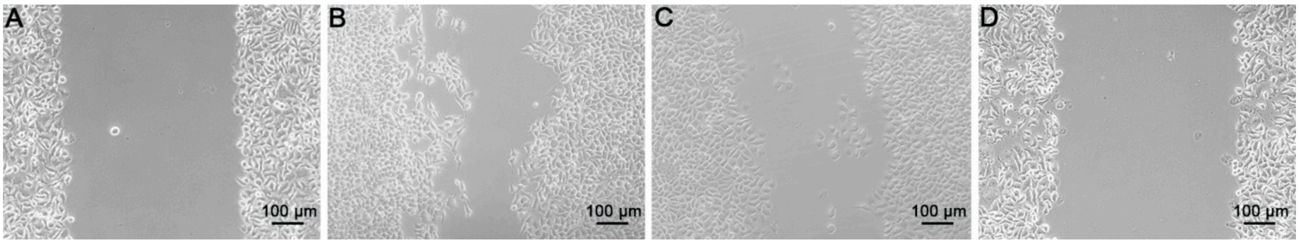

Figure S9. Effect of **PD4** treatment on the migration of HepG2 cancer cells. Photographs of HepG2 cells at (**A**) 0 h, (**B**) 24 h without treatment, (**C**) 24 h with 100 µg/mL **PD4** treatment at pH 7.4, and (**D**) 24 h with 0.25 × IC_{50} (5 µg/mL) of **PD4** treatment at pH 6.5.

Table S1. Degree of polymerization (DP) and molecular weights of PZLO-*b*-PLF.

Polymer	DP of PZLOn-*b*-PLFm				Molecular weight (kDa)		
	n_{calc}	m_{cal}	n_{NMR}	m_{NMR}	Calc.	H NMR	GPC (PDI)
**PZ1**	30	0	30.4	0	7.48	7.64	No test
**PZ3**	30	4	30.4	4.09	8.07	8.24	8.33 (1.73)
**PZ3**	30	8	29.2	7.91	8.66	8.51	8.97 (1.63)
**PZ4**	30	12	29.1	11.3	9.24	8.98	9.43 (1.59)

References

1. Pan, M.; Lu, C.; Zheng, M.; Zhou, W.; Song, F.; Chen, W.; Yao, F.; Liu, D.; Cai, J., Unnatural amino acid-based star-shaped poly(L-ornithine)s as emerging long-term and biofilm-disrupting antimicrobial peptides to treat Pseudomonas aeruginosa infected burn wounds. *Adv. Healthc. Mater.* **2020**, *9*, e2000647.
2. Zheng, M.; Lin, H.; Zhang, W.; Tang, S.; Liu, D.; Cai, J., Poly(L-ornithine)-grafted zinc phthalocyanines as dual-functional antimicrobial agents with intrinsic membrane damage and photothermal ablation capacity. *ACS Infect. Dis.* **2021**, 7, 2917–2929.
3. Maeda, Y.; Pittella, F.; Nomoto, T.; Takemoto, H.; Nishiyama, N.; Miyata, K.; Kataoka, K., Fine-tuning of charge-conversion polymer structure for efficient endosomal escape of siRNA-loaded calcium phosphate hybrid micelles. *Macromol. Rapid Commun.*, **2014**, *35*, 1211–1215.
